# Seasonal influenza vaccination in patients with COPD: a systematic literature review

**DOI:** 10.1186/s12890-017-0420-8

**Published:** 2017-05-03

**Authors:** Rafik Bekkat-Berkani, Tom Wilkinson, Philippe Buchy, Gael Dos Santos, Dimitris Stefanidis, Jeanne-Marie Devaster, Nadia Meyer

**Affiliations:** 1 GSK, Wavre, Belgium; 2 Clinical and Experimental Sciences, Faculty of Medicine, University of Southampton, Southampton, UK; 3 GSK, Singapore, Singapore; 4 Business & Decision Life Sciences, Brussels, Belgium; 5 GSK, Rixensart, Belgium; 6 Present address: GSK, Wavre, Belgium; 7 Present address: Boehringer-Ingelheim Pharma GmbH & Co. KG, Ingelheim, Germany; 8 Present address: GSK, 5 Crescent Drive, Philadelphia, PA 19112 USA

**Keywords:** Influenza, Vaccination, COPD, Immunogenicity, Efficacy, Effectiveness, Systematic review

## Abstract

**Background:**

Influenza is a frequent cause of exacerbations of chronic obstructive pulmonary disease (COPD). Exacerbations are associated with worsening of the airflow obstruction, hospitalisation, reduced quality of life, disease progression, death, and ultimately, substantial healthcare-related costs. Despite longstanding recommendations to vaccinate vulnerable high-risk groups against seasonal influenza, including patients with COPD, vaccination rates remain sub-optimal in this population.

**Methods:**

We conducted a systematic review to summarise current evidence from randomised controlled trials (RCTs) and observational studies on the immunogenicity, safety, efficacy, and effectiveness of seasonal influenza vaccination in patients with COPD. The selection of relevant articles was based on a three-step selection procedure according to predefined inclusion and exclusion criteria. The search yielded 650 unique hits of which 48 eligible articles were screened in full-text.

**Results:**

Seventeen articles describing 13 different studies were found to be pertinent to this review. Results of four RCTs and one observational study demonstrate that seasonal influenza vaccination is immunogenic in patients with COPD. Two studies assessed the occurrence of COPD exacerbations 14 days after influenza vaccination and found no evidence of an increased risk of exacerbation. Three RCTs showed no significant difference in the occurrence of systemic effects between groups receiving influenza vaccine or placebo. Six out of seven studies on vaccine efficacy or effectiveness indicated long-term benefits of seasonal influenza vaccination, such as reduced number of exacerbations, reduced hospitalisations and outpatient visits, and decreased all-cause and respiratory mortality.

**Conclusions:**

Additional large and well-designed observational studies would contribute to understanding the impact of disease severity and patient characteristics on the response to influenza vaccination. Overall, the evidence supports a positive benefit-risk ratio for seasonal influenza vaccination in patients with COPD, and supports current vaccination recommendations in this population.

**Electronic supplementary material:**

The online version of this article (doi:10.1186/s12890-017-0420-8) contains supplementary material, which is available to authorized users.

## Background

Chronic obstructive pulmonary disease (COPD) is a major cause of morbidity and mortality with a significant disease burden in both primary and secondary care. In 2010, 384 million individuals worldwide were estimated to have COPD, with a global prevalence of 11.7% [1]. COPD is the most common cause of death due to chronic respiratory disease, with 2.9 million deaths estimated in 2013 [2]. COPD ranks as the third most common cause of death in the United States (US) and fourth in the United Kingdom (UK) and Southern Latin America [2]. The prevalence of COPD increases significantly with age and tobacco use, and is higher in men than in women [3].

There is no known cure for COPD, but the symptoms are treatable and disease progression can be delayed [4]. The frequency and severity of COPD exacerbations is strongly linked to disease progression, quality of life, hospitalisation, morbidity and mortality [5]. Exacerbations of COPD are characterised by acute worsening of symptoms due to airflow restriction resulting from mucus hypersecretion, mucosal swelling and bronchospasm. It is estimated that the cost of managing COPD exacerbations accounts for 40% of the total cost of COPD, with a substantial portion attributable to hospitalisations [6].

At least 70% of COPD exacerbations are infectious in origin, with respiratory viruses identified in approximately 30% of cases [7, 8]. In a review of the literature, influenza was the second most common virus identified associated with COPD exacerbations, with a prevalence ranging from 2.5 to 11.6%, the first one being the rhinovirus (prevalence 7.2 to 27.3%) [8]. Bacterial and viral co-infections may also occur, and bacterial infection may complicate an initial viral infection.

In view of the role of influenza in contributing to COPD exacerbations, the associated complications and their related healthcare costs, immunisation against influenza is recommended for all patients with COPD by the World Health Organization (WHO), the US Centers for Disease Control and Prevention, the European Centre for Disease Control and Prevention (ECDC), and numerous national agencies [9–11]. Descriptive population-based and cohort studies have shown that influenza vaccination significantly reduces hospitalisations and mortality in patients with COPD [12–14]. Nevertheless, influenza vaccination coverage rates remain below target in many countries. US 2014–15 coverage of high-risk adults between 18 and 64 years with influenza vaccine was 47.6%, and 66.7% in adults ≥ 65 years, both below the target of 70% [15]. The European target for vaccine coverage among individuals with chronic medical conditions is 75%. In 2012–13 median influenza vaccine coverage for this population was 45.6%, ranging from 28.0 to 80.2% across the 7 reporting countries [9].

The currently available seasonal influenza vaccines are either trivalent vaccines (TIVs) containing one strain of each of the two subtypes of influenza A virus (A/H1N1 and A/H3N2) and one of the two co-circulating B-virus lineages (B/Victoria or B/Yamagata), or (since 2012 in several countries) quadrivalent vaccines (QIVs) containing both influenza A subtypes mentioned above, and both influenza B co-circulating lineages (B/Victoria and B/Yamagata) [16]. QIVs are expected to provide broader protection than TIVs against co-circulation of influenza type B viruses that occurs each season, and in seasons characterised by either an unpredicted mismatch between the influenza B lineage contained in the vaccine and the predominantly circulating type B lineage [16, 17].

A Cochrane literature review of randomised controlled trials (RCTs) published by Poole et al., in 2006 concluded that influenza vaccination appears to reduce exacerbations of COPD, although this was based on a limited number of reports [18]. An update of the Cochrane review in 2010 did not result in additional studies. We conducted an updated systematic literature review to summarise the current evidence from RCTs and observational studies on the immunogenicity, safety, efficacy and effectiveness, quality of life and preventable treatment costs of seasonal influenza vaccination in patients with COPD. The aim of the review is to inform healthcare professionals on the reported risks and benefits of seasonal influenza vaccination in patients with COPD.

## Methods

The systematic literature review followed Cochrane guidelines and Preferred Reporting Items for Systematic Reviews and Meta-Analysis (PRISMA) guidelines [19, 20].

### Search strategy

The literature search was performed in the PubMed, Embase and Cochrane Library databases from January 1^st^ 1990 to September 15^th^ 2015. Search strings combining terms for “COPD”, “influenza”, and “vaccination” were used. The complete search strategy is provided in the Additional file 1. No geographic or language limitation was applied.

### Inclusion and exclusion criteria

Databases were searched for publications on individuals with COPD and influenza vaccination. RCTs and observational studies describing the immunogenicity, safety, efficacy and effectiveness, quality of life and preventable treatment costs of seasonal influenza vaccination on COPD outcomes were included. Articles had to include data relevant to the objectives. The following studies were excluded: 1) Studies including patient groups with a mix of pulmonary diseases if results were not presented for COPD separately; 2) Studies with co-administration of pneumococcal vaccine to avoid confounding vaccine effects; 3) Efficacy studies without a control group receiving placebo; 4) Studies evaluating pandemic influenza vaccines only; 5) Letters to the editor, editorials, case reports or comments; 6) Articles published in languages other than English, Spanish, Italian, French, Dutch or German; 7) Studies of insufficient methodological quality (as determined below); 8) Studies with mixed results for adults and children with no data presented separately; 9) Modelling studies.

### Study selection and critical appraisal

Articles were selected by a three-step selection procedure based on 1) screening of title and abstract, 2) screening of full-text article, and 3) final screening during the data-extraction phase. Titles and abstracts retrieved from the three databases (the PubMed, Embase and Cochrane Library) were screened in duplicate by two researchers independently from each other. The results were compared and discussed; all selected references from the two researchers were included for full text selection. The first 10% of the full text articles was critically appraised in duplicate by two independent researchers. In case of discrepancy or disagreements, a third researcher was consulted. If multiple articles reported on the same study, only the most relevant or most comprehensive article was included in this review. The methodological quality of each of the included studies was evaluated using checklists from the Scottish Intercollegiate Guidelines Network [21].

#### Data extraction

Data were extracted into pre-defined evidence tables containing information on study characteristics (country, design, influenza season, follow-up period and setting); study population (inclusion and exclusion criteria, age, gender and case definition); study results and critical appraisal. Four articles were excluded, because they did not provide additional information to the articles included in this systematic review [22–25]. Data are presented as expressed in the original study, no recalculations were done. Because of the diverse range of study designs and many different outcome measures results could not be summarised in a meta-analysis.

#### Definitions

Strain-specific immune responses to the influenza virus haemagglutinin surface glycoprotein measured by a haemagglutination inhibition (HI) test are widely accepted indicators of immunogenicity. Seroconversion is at least a 4-fold increase in serum HI titre post-vaccination compared with baseline. The seroconversion rate in a population is considered sufficient (meeting predefined criteria for licensure) for seasonal influenza vaccine by the European Committee for Medicinal Products for Human Use (CHMP) when above 40% in subjects aged 18–60 years and above 30% in subjects older than 60 years. The acceptable seroconversion rate for the US Center for Biologics Evaluation and Research (CBER) is when the lower limit of the 95% confidence interval (CI) is at least 40% in subjects aged 18–60 years and at least 30% in subjects older than 60 years [16, 26, 27].

The seroprotection rate is defined as the proportion of the population with HI titres ≥1:40 at four weeks post-vaccination. The seroprotection rate in a population is considered sufficient for seasonal influenza vaccines by CHMP when above 70% in subjects aged 18–60 years and above 60% in subjects older than 60 years, and by CBER when the lower limit of the 95% CI is at least 70% in 18–60 year olds and at least 60% in subjects older than 60 years [16, 26, 27].

Safety of vaccination was studied by comparing local and systemic effects of influenza and placebo vaccination. COPD exacerbations up to 14 days post-vaccination were considered a possible adverse effect of vaccination. The term reactogenicity refers to adverse events that are common and known to occur after vaccination and should be registered [28].

Vaccine efficacy is commonly defined as the direct effect of a vaccine measured in pre-licensure randomised clinical trials where vaccination is allocated under optimal conditions, comparing a vaccinated group with a placebo group in the same population. Vaccine effectiveness is a “real world” view of how a vaccine works under field conditions in a population once the vaccine is marketed [29, 30].

## Results

**Fig. 1:**
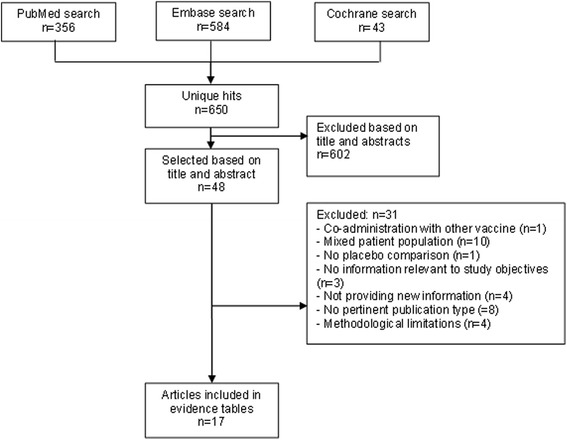
Selection of studies: databases used and criteria for exclusion (*n* = number of studies)

The search yielded 650 unique hits of which 48 eligible articles were screened in full text. Seventeen articles describing 13 different studies were found to be pertinent to this review (Fig. 1).

### Characteristics of included studies

Table 1 shows the characteristics of the included studies. There were four RCTs reported in seven articles, and nine observational studies that included two prospective cohort studies, five retrospective cohort studies (reported in six articles), and two self-controlled case series (Table 1). The studies were performed across different influenza seasons in Australia, India, Spain, Taiwan, Thailand, UK and the US with sample sizes ranging from 29 to 40,741 subjects. The main risks of study bias are described in Table 1, the most frequent being self-reported outcomes, lack of *a priori* evaluations of study statistical power, retrospective design and limited information on exposure or outcome assessment. Seven of the 13 studies were assessed as being of acceptable quality (Table 1).

**Table 1:** Characteristics of included studies, patient populations, topic and results of risk of bias assessment

Reference (Country)	Study design (population)	Influenza season (setting)	Mean (SD) Age^a^	Gender, % male^a^	N total	Outcome	Potential bias identified & expected impact (Quality assessment^b^)
Randomised controlled trials							
Chuaychoo et al., 2010 [31] (Thailand)	Open label RCT (Patients with confirmed COPD)	2006–2007 (1 hospital COPD clinic)	73 (9)	90.7%	75	Immunogenicity Safety	No placebo-controlled group; open-label design; self-reported safety outcomes (LOW)
Gorse et al., 2004^c^ [32] (US)	Placebo-controlled RCT (Patients ≥ 50 years with confirmed COPD)	1998–1999 (20 Veterans affairs medical centres)	67.9 (8.5)/67.8 (8.2)	98.0/98.4%	2,215	Immunogenicity	Serum titres tested in 61 subject; no placebo-controlled/unvaccinated group (ACCEPTABLE)
Gorse et al., 2003^c^ [39]					2,215	Safety	Self-reported safety outcomes (ACCEPTABLE)
Kositanont et al., 2004^d^ [40] (Thailand)	Placebo-controlled RCT (2 doses 1 month apart) (Patients with confirmed COPD)	1997–1998 (1 hospital COPD clinic)	67.6 (8)/69.1 (7)	95.2/93.7%	123	Immunogenicity Efficacy	Self-reported safety outcomes (ACCEPTABLE)
Wongsurakiat et al. 2004a^d^[35]					125	Immunogenicity Efficacy	
Wongsurakiat et al., 2004b^d^[38]					125	Safety	
Gorse et al., 1997 [33] (US)	RCT (Outpatients with confirmed COPD)	(Veterans affairs medical centres)	65.2 (2.1)	100%	29	Immunogenicity Safety	Small sample size; no estimate of study power; limited information on patient characteristics; males only (LOW)
Observational studies							
Nath et al., 2014 [34] (Australia)	Prospective cohort study (Patients with confirmed COPD)	2010 (outpatient clinics 1 hospital)	66.2 (11.0)/54.3 (14.5)	65.0/57.1%	34	Immunogenicity	Small sample size; no estimate of study power; significant differences between groups at baseline; serological response to only one vaccine strain (LOW)
Chen et al., 2013^e^ [45] (Taiwan)	Retrospective, Database study (Patients ≥ 55 years with COPD diagnosed using ICD-9 codes)	2000–2007 (Taiwan National Health Insurance Research Dataset)	-	58.9/53.4%	25,609	Effectiveness	Retrospective design; no estimate of study power (ACCEPTABLE)
Sung et al., 2014^e^ [46]			≥55	58.7/60.8%	7,722	Effectiveness	Retrospective design; estimate of study power (ACCEPTABLE)
Menon et al., 2008 [43] (India)	Self-controlled case series (Male patients with confirmed COPD)	2004–2006 (outpatient department 1 hospital)	64.8 (8)	100%	87	Effectiveness	Small sample size; no estimate of study power; males only; comparison of different influenza seasons (LOW)
Schembri et al., 2009 [42] (UK)	Retrospective, database study (Patients ≥ 40 year registered in the data base with COPD)	1988–2006 (The Health Improvement Network)	-	42.4/42.8%	40,741	Effectiveness	Retrospective design; no estimate of study power (ACCEPTABLE)
Vila-Córcoles et al., 2008 [41] (Spain)	Prospective cohort study (Community-dwelling ≥65 years olds registered with COPD in clinic record)	2002–2005 (8 urban health centres)	74.1 (6.8)/76.3 (6.9)	73.7/74.5%	1,298	Effectiveness	Significant differences between groups at baseline; no estimate of study power (ACCEPTABLE)
Ting et al., 2011 [36] (UK)	Retrospective matched cohort study (Patients with confirmed COPD)	2005 (6 general practices)	68 (37–89)	64.8%	586	Safety	Retrospective design; sample size calculations performed but details not presented; limited information on patient characteristics; matched pairs of patients, but no results of matching (LOW)
Montserrat-Capdevila et al., 2014 [44] (Spain)	Retrospective cohort study (Patients registered with confirmed COPD)	2001–2002 (1 hospital)	75.6 (11.7)/57.1 (18.2)	65.2/60.5%	1,323	Effectiveness	Retrospective design; no estimate of study power (ACCEPTABLE)
Tata et al., 2003 [37] (UK)	Database study with self-controlled case series (Random sample of patients with COPD using OXMIS and READ codes)	1991–1994 (Clinical Practice Research Datalink, previously General Practice Research Database)	65–79	63%	2,100	Safety	Retrospective design; limited information on patient characteristics (ACCEPTABLE)
Wang et al., 2003 [12] (Taiwan)	Retrospective population-based cohort study (>65 years old patients with COPD identified using ICD-9 mortality codes)	2001	No data for patients with COPD		102,698 elderly	Effectiveness	Retrospective database design; no estimate of study power; COPD identified from mortality ICD-9 codes; Reason for risk status not known; limited information on patient characteristics (LOW)

*N* number of subjects, *RCT* randomised controlled trial, *SD* standard deviation, *UK* United Kingdom, *US* United States, *COPD* chronic obstructive pulmonary disease, *ICD-9* international classification of disease version 9. Confirmed COPD, *COPD* confirmed according to spirometry criteria

^a^Age and gender presented as vaccinated/unvaccinated or controls

^b^Study quality according to Scottish Intercollegiate Guidelines Network (SIGN) checklists [21]. Studies classified as of low or acceptable quality

^c^Gorse et al., 2004 and Gorse et al., 2003 describe the same RCT

^d^Kositanont et al., 2004, Wongsurakiat et al., 2004a and Wongsurakiat et al., 2004b describe the same RCT, with minor discrepancies in patient characteristics

^e^In the study of Sung et al., 2014 a subpopulation of Chen et al., 2013 is used

### Immunogenicity of seasonal influenza vaccines

**Fig. 2:**
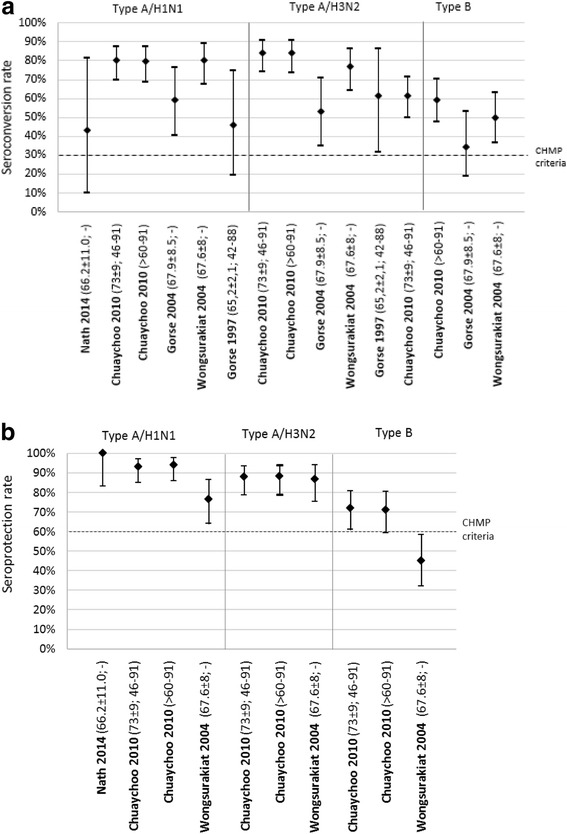
**a** Seroconversion rate (95% Confidence interval) 4 weeks after seasonal influenza vaccination in patients with COPD (reference [mean age ± SD; range]), European Committee for Medicinal Products for Human Use (CHMP) criteria for licensure for subjects aged >60 years. **b** Seroprotection rate (95% Confidence interval) 4 weeks after seasonal influenza vaccination in patients with COPD (reference [age ± SD; range]), European Committee for Medicinal Products for Human Use (CHMP) criteria for licensure for subjects aged >60 years

Five studies (four RCTs and one observational study) assessed the immunogenicity of TIVs in patients with COPD (Table 2 and Fig. 2a and b) [31–35]. The seroconversion rates ranged from 43 to 80.0% for A/H1N1, 53.1 to 84.1% for A/H3N2 and 34.4 to 61.3% for influenza B. [26, 27]. Predefined CHMP criteria, but not CBER criteria, used for licensure of seasonal influenza vaccines were met in all studies except for influenza type B in Gorse et al. [32] in which all subjects were ≥ 50 years of age. In this study, the CHMP criteria were met for influenza B considering the criteria applicable to the >60 year age group.

**Table 2:** Seroconversion and seroprotection rates 4 weeks after seasonal influenza vaccination in patients with COPD

Reference	Country	Influenza season	Vaccine type	Influenza type/subtype	Immunogenicity outcome	
					Percentage with ≥4-fold increase of antibody titres (95% CI) (n/N)	Percentage with antibody titre ≥1:40 (95% CI) (n/N)
Nath et al., 2014 [34]	Australia	2010^a^	TIV	Type A/H1N1	43% (9.9–81.6) (3/7)	100% (83.2–100) (20/20)
Chuaychoo et al., 2010 [31]	Thailand	2006–2007	TIV	*All subjects*		
				Type A/H1N1	80.0% (69.6–87.5) (60/75)	93.3% (85.1–97.1) (70/75)
				Type A/H3N2	84.0% (74.1–90.6) (63/75)	88.0% (78.7–93.6) (66/75)
				Type B	61.3% (50.0–71.5) (46/75)	72.0% (61.0–80.9) (54/75)
				*Subjects > 60 year*		
				Type A/H1N1	79.7% (68.8–87.5) (55/69)	94.2% (86.0–97.7) (65/69)
				Type A/H3N2	84.1% (73.7–90.9) (58/69)	88.4% (78.8–94.0) (61/69)
				Type B	59.4% (47.6–70.2) (41/69)	71.0% (59.4–80.4) (49/69)
Gorse et al., 2004 [32]	US	1998–1999	TIV + placebo	Type A/H1N1	59.4% (40.6–76.3) (19/32)	-
				Type A/H3N2	53.1% (34.7–70.9) (17/32)	-
				Type B	34.4% (18.6–53.2) (11/32)	-
Wongsurakiat et al., 2004 [35]	Thailand	1997–1998	TIV (2 doses)^b^	Type A/H1N1	80.0% (67.7–89.2) (48/60)‡	76.6% (64.0–86.6) (46/60)
				Type A/H3N2	76.7% (64.0–86.6) (46/60)‡	86.7% (75.4–94.1) (52/60)
				Type B	50.0% (36.8–63.2) (30/60)‡	45.0% (32.1–58.4) (27/60)
Gorse et al., 1997 [33]	US	1994–1995	TIV + placebo	Type A/H1N1	46.2% (19.2–74.9) (6/13)	-
				Type A/H3N2	61.5% (31.6–86.1) (8/13)	-

*CAIV-T* cold-adapted influenza virus, *CAV* cold-adapted influenza virus, *n* number of subjects, *RCT* Randomised controlled trial, *TIV* trivalent influenza vaccine, *US* United States, *n/N* number of subjects with a response/HI titre ≥ 1:40 over the total number of subjects, *95% CI* 95% confidence intervals. Exact 95% CI calculated when not provided in the article

^a^Study conducted in Australia: influenza season from April 2010 to November 2010

^b^Results after dose 1 are shown. 4-fold increases after the second dose were low

-not reported

In three studies in which seroprotection rates were measured four weeks after vaccination, at least 76.6% of subjects had titres ≥ 1:40 for influenza A subtypes and 45.0 to 72.0% had titres ≥ 1:40 for influenza B. Predefined CHMP and CBER licensure criteria were met in all studies for influenza type A, except for influenza A/H1N1 in one study conducted in Thailand [35]. Predefined CHMP criteria were met for influenza B in two out of three studies, but were not met using CBER criteria.

### Safety and reactogenicity of seasonal influenza vaccines

Two studies assessed the occurrence of COPD exacerbations after influenza vaccination compared to either unvaccinated controls [36], or using a self-controlled method consisting of comparing a pre-defined vaccination period with other time periods in the influenza season for each vaccinated subject [37]. Neither study found evidence of an increased risk of exacerbation up to 14 days post-vaccination (Additional file 2). In one small RCT conducted in 29 subjects, spirometry results of a group receiving TIV plus trivalent live, cold-adapted influenza virus vaccine were compared with a group receiving TIV plus placebo. Spirometry did not change significantly after vaccination, and did not differ between treatment groups (Additional file 2) [33].

Two RCTs recorded local symptoms occurring at the vaccine injection sites [31, 38] (Additional file 2). Pain at the site of injection was the most frequently mentioned local reaction after vaccination. Other local reactions were itching, erythema and swelling. In one RCT that compared influenza vaccine with placebo, local reactions were recorded significantly more frequently after administration of the vaccine than after administration of placebo [38].

Three RCTs recorded the systemic effects of seasonal influenza vaccination (Additional file 2) [31, 38, 39]. The most frequently occurring systemic effects were myalgia, headache, fever and dyspnoea. There was no significant difference in the occurrence of systemic effects between group receiving influenza vaccine or placebo [38, 39].

### Efficacy of seasonal influenza vaccines

Only one placebo-controlled RCT (reported in two articles) described the efficacy of two doses of seasonal influenza vaccination in 125 previously unvaccinated patients with COPD [35, 40] (Table 3). After one year of follow-up, vaccinated patients experienced significantly (*p* = 0.005) fewer episodes of influenza-related acute respiratory illness (ARI) than unvaccinated patients, respectively four of 62 patients versus 17 of 63 patients. Vaccine efficacy of influenza vaccination in preventing ARI was 76% (Risk ratio [RR] 0.2, 95% confidence interval [CI] 0.06–0.7). Vaccinated patients also had significantly fewer outpatient visits episodes, respectively two of 62 versus 12 of 63 (*p* = 0.009) (Table 3) [35]. There was no statistically significant difference between vaccinated and unvaccinated in the number of hospitalisations (two in vaccinated and five in unvaccinated patients) or episodes of mechanical ventilation (none in vaccinated patients and three in unvaccinated patients). An analysis of the clinical presentation of ARI in vaccinated and unvaccinated patients showed that the incidence of common cold and acute exacerbation did not differ between groups, however vaccinated patients had significantly less influenza-like illness than unvaccinated patients (5 episodes versus 15 episodes, respectively) [35]. HI antibody titres after vaccination showed that the confirmed influenza cases in the vaccinated group were non-responders to vaccination [40].

**Table 3:** Efficacy outcomes after one year of follow-up after seasonal influenza vaccination in patients with COPD

Reference	Country	Study design	Influenza season	Vaccine type	n	Efficacy outcome	Comment
Kositanont et al., 2004 [40]	Thailand	RCT	1997–1998^a^			*ARI with confirmed influenza (incidence (n/N))*	Study conducted in non-epidemic years. Most circulating A/H3N2 viruses among patients with acute respiratory infections matched the vaccine strain.
				TIV	61	8.2% (5/61)	
				Placebo	62	27.4% (17/62)	
Wongsurakiat et al., 2004 [35]	Thailand	RCT	1997–1998^a^			*IR of influenza-related ARI episodes*	
				TIV	62	6.8 per 100 py	
				Placebo	63	28.1 per 100 py (*p* = 0.0005)	
						*IR of outpatient episodes*	
				TIV	62	3.4 per 100 py	
				Placebo	63	19.8 per 100 py (*p* = 0.009)	
						*IR of hospitalisation episode*	
				TIV	62	3.4 per 100 py	
				Placebo	63	8.3 per 100 py (*p* = 0.3)	
						*IR of mechanical ventilation episode*	
				TIV	62	0	
				Placebo	63	5.0 per 100 py (*p* = 0.1)	

*ARI* acute respiratory illness, *IR* incidence rate, *n* number of subjects, *py* person years, *RCT* randomised controlled trail, *TIV* trivalent iinfluenza vaccine, *Placebo* Vitamin B1 injection, *n/N* number of subjects with the outcome indicated over the total number of subjects

Wongsurakiat et al., 2004 and Kositanont et al., 2004 describe the same RCT

^a^Enrolment between June 1997 – November 1998, subjects were followed for one year after vaccination

### Effectiveness of the seasonal influenza vaccine

Three observational studies described all-cause mortality after seasonal influenza vaccination [12, 41, 42] (Table 4). In the prospective Spanish cohort study by Vila-Córcoles et al., [41] of 1,298 subjects with COPD, seasonal influenza vaccination did not reduce the risk of all-cause mortality each year or overall during the four-year follow-up period (Hazard Ratio 0.76, 95% CI 0.52–1.06) [41]. Similarly, in a retrospective cohort study, influenza vaccination was not associated with a statistically significant reduction in the risk of all-cause death in the year following immunisation (Odds Ratio 0.76, 95% CI 0.41–1.40) [14]. By contrast, a retrospective study in the UK using The Health Improvement Network (THIN) which included data from almost 41,000 patients with COPD, showed a protective effect of seasonal influenza vaccination [42]. Over an average 6.8 year follow-up period between 1988 and 2006, influenza vaccination was associated with a reduced risk of all-cause mortality by 41% (RR 0.59 [95% CI 0.57–0.61]) [42]. Wang et al. [12] also found that influenza vaccination was associated with significantly reduced mortality due to COPD as identified by ICD-9 codes, in more than 102,000 elderly individuals ≥ 65 years considered at low risk and at high risk (recent hospital admission or chronic disease) for severe influenza (Table 4).

**Table 4:** Effectiveness outcomes, mortality and hospitalisation, after seasonal influenza vaccination in COPD patients

Reference (Country)	Study design	Influenza season	n	Subgroup analysis	Effectiveness outcome	Comment
					*Mortality*	
Schembri et al., 2009 [42] (UK)	Database study	1988–2006^a^	40,741		*RR (95% CI) all-cause* 0.59 (0.57–0.61)	Mortality rates were higher in years when the influenza vaccine did not include all strains circulating during that season (RR 1.19, 95% CI 1.13–1.25).
					*RR (95% CI) death associated with respiratory event* 0.63 (0.58–0.68)	
					*RR (95% CI) with respiratory event recorded as cause of death* 0.63 (0.55–0.77)	
Vila-Córcoles et al., 2008 [41] (Spain)	Prospective cohort study				*HR (95% CI) all-cause*	Mild-moderate influenza activity during the study. Mixed circulation of influenza A and B, with generally good matches with vaccine strains
		2002	1,298		0.48 (0.22–1.04)	
		2003	1,233		0.79 (0.37–1.60)	
		2004	1,149		0.95 (0.48–2.03)	
		2005	1,050		0.87 (0.43–1.77)	
		All seasons	-		0.76 (0.52–1.06)	
Wang et al., [12] (Taiwan)	Retrospective population-based cohort study	2001	102,698 elderly		*RR (95% CI) with COPD recorded as cause of death*	Good match between epidemic strains and vaccine strains [55]
				High-risk^b^	0.45 (0.32–0.63)	
				Low-risk	0.47 (0.26–0.83)	
					*Hospitalisation*	
Chen et al., 2013^c^ [45] (Taiwan)	Retrospective, Database study	2000–2007^a^		*Gender*	*HR (95% CI) due to heart failure*	Good match between epidemic strains and vaccine strains except for 2001–02 (B mismatch), 2003–04 (A/H3N2 mismatch) [55]
			11,749	Female	0.48 (0.33–0.68)	
			13,860	Male	0.42 (0.32–0.57)	
				*Age groups*	*HR (95% CI) due to heart failure*	
			13,218	≤44 years	3.96 (0.50–31.11)	
			4,669	45–54 years	2.67 (0.95–7.50)	
			3,455	55–64 years	0.65 (0.38–1.10)	
			2,854	65–74 years	0.37 (0.26–0.52)	
			1,413	≥75 years	0.38 (0.26–0.55)	
			25,609	All subjects	0.44 (0.35–0.55)	
Sung et al., 2014^c^ [46] (Taiwan)	Retrospective, Database study	2000–2007^a^	7,722	≥ 55 years	*HR (95% CI) due to acute coronary syndrome*	As above
				Influenza season	0.45 (0.35–0.57)	
				Non-influenza season	0.48 (0.37–0.62)	
				All seasons	0.46 (0.39–0.55)	
Menon et al., 2008 [43] (India, New Delhi)	Self-controlled case series	2004–2006		*COPD severity*	*RR (p-value) post-vaccination year compared to pre-vaccination year* *Hospitalisation*	Poorly matched seasons in 2005 and 2006 for influenza A strains (data for Kolkata) [56].
			32	Mild	0.33 (0.31)	
			17	Moderate	0.5 (0.41)	
			38	Severe	0.14 (0.15)	
			87	Total	0.28 (0.02)	
					*ARI*	
			32	Mild	0.4 (0.26)	
			17	Moderate	0.4 (0.21)	
			38	Severe	0.25 (0.02)	
			87	Total	0.33 (0.005)	
Montserrat-Capdevila et al., 2014 [44] (Spain)	Retrospective cohort study	2011–2012		*COPD severity*	*OR (95% CI) due to COPD exacerbations*	Moderately severe influenza season. Moderate-to good matches for predominant circulating A/H1N1 and A/H3N2 viruses. Poor match for type B [14]
			1,099	Mild	0.083 (0.042–0.163)	
			108	Moderate	0.133 (0.021–0.844)	
			62	Severe	0.305 (0.024–3.813)	
			54	Very severe	0.067 (0.009–0.505)	
			1,323	Total	0.092 (0.052–0.165)	

*CI* confidence interval, *COPD* chronic obstructive disease, *HR* hazard ratio, *n* number of subjects, *OR* odds ratio, *RR* relative risk, *UK* United Kingdom

Disease severity by Menon et al., 2008: mild: FEV_1_ > 70% predicted; moderate: FEV_1_ = 50–69% predicted; severe: FEV_1_ < 50% predicted. Disease severity by Montserrat-Capdevila et al., 2014: mild: FEV_1_ > 80% predicted; moderate: FEV_1_ = 50–80% predicted; severe: FEV_1_30–50% predicted; very severe: FEV_1_ < 30% predicated

^a^Data from different influenza seasons were not separately analysed

^b^High risk defined as recent hospital admission or chronic disease

^c^In the study of Sung et al., 2014 a subpopulation of Chen et al., 2014 is used

Four observational studies of varying size (three of retrospective cohort design and one self-controlled case-series) described hospitalisation episodes in patients with COPD after seasonal influenza vaccination [43–46] (Table 4). A retrospective study of more than 25,000 individuals using the Taiwan National Health Insurance Research Dataset (2000–2007) showed an associated benefit of seasonal influenza vaccination in elderly patients (≥65 years, *p* < 0.05) but not in younger age groups, on the hospitalisation rate attributable to heart failure [45]. A sub-analysis of patients with COPD who were aged ≥55 years showed that the hazard ratio of hospitalisation due to acute coronary syndrome was significantly lower (*p* < 0.001) in vaccinated individuals than unvaccinated individuals over eight years of follow-up [46]. This study also demonstrated a significant benefit (*p* < 0.001) in repeating influenza vaccinations in the same patient across several study seasons; four or more vaccinations over eight influenza seasons resulted in a substantial reduction in hospitalisations due to acute coronary syndrome in patients with COPD.

Menon et al., [43] assessed 87 Indian patients one year before and one year after influenza vaccination (2004–2006) using a self-controlled case series study design. Seasonal influenza vaccination was associated with a reduction in the risk of hospitalisation in the overall COPD patient population (*p* = 0.02), but not when groups were further classified by illness severity: i.e., with mild, moderate or severe disease [43]. ARI occurred significantly more frequently in the pre-vaccination year than in the post-vaccination year (27.6% [24/87] versus 9.2% [8/87], *p* = 0.005). Hospitalisations also occurred significantly more frequently in the pre-vaccination than in the post-vaccination year (16.1% [14/87] versus 4.6% [4/87], *p* = 0.02). Visits to the outpatient department and number of patients who required mechanical ventilation did not differ between pre- and post-vaccination years.

Montserrat-Capdevila et al., [44] studied the risk of hospitalisation due to exacerbations in 1,323 vaccinated (mean age 75.6 years) and unvaccinated (mean age 57.1 years) Spanish patients with COPD during the 2001–2002 influenza season. The odds ratio (OR) (95% CI) for the risk of hospitalisation due to COPD exacerbations for vaccination compared with no vaccination was 0.092 (0.052–0.165). The effectiveness of influenza vaccination in preventing hospitalisation was 90.8% (95% CI 83.5–94.8).

### Quality of life measures or treatment costs

Our search returned no studies describing the impact of influenza vaccination on quality of life measures or treatment costs for patients with COPD. However, an economic evaluation was found to have been conducted in Thailand based on the results of the randomised controlled efficacy study described above [35, 40]. The authors noted that the majority (90%) of the cost of treating COPD exacerbations was attributable to hospitalisation. They concluded that influenza immunisation was cost-effective in patients with COPD, with a greater cost-benefit in those with more severe underlying disease [47].

## Discussion

Ten years after Poole et al., [18] reported a systematic review of RCTs that examined influenza vaccination in patients with COPD, we identified only one new RCT contributing to this body of data. The paucity of studies is not unexpected in view of well-established recommendations to immunise patients with COPD against influenza, making placebo-controlled trials ethically questionable and thus difficult to conduct. Indeed, the only RCT we identified since the review by Poole et al., was an open label study to assess the immunogenicity of two different influenza vaccines [31]. Unlike Poole et al., we did not include RCTs conducted in mixed patient populations with cardiac or pulmonary conditions or elderly with chronic diseases. Three RCTs (four references) were included in both reviews [33, 35, 38, 39]. Nevertheless, the conclusions of the reviews of RCTs are comparable: that immunisation with seasonal influenza vaccines appears to reduce acute exacerbations in patients with COPD. In the current setting where placebo-controlled clinical trials are not possible to conduct, observational studies play an important role in describing the real-world impacts of vaccination in specific populations, and better reflect the true impact of vaccination during the post-licensure phase. For this reason, we included observational studies in our review, and identified seven studies that evaluated immunogenicity, safety or effectiveness of influenza vaccination in patients with COPD. We also built on the review of Poole et al., by reviewing the immunogenicity of seasonal influenza vaccines in patients with COPD.

An impaired immune response to vaccination and infection in patients with COPD has been described [34, 48]. Immunogenicity in this population may also be influenced by immune senescence in older adults, comorbidities, and the use of immunosuppressants. Influenza vaccination was immunogenic in patients with COPD in the five studies that reported on vaccine immunogenicity [31–35]. High levels of seroprotection were achieved in all studies for most vaccine strains and CHMP criteria indicating acceptable immunogenicity levels were achieved in most studies. However, further studies which identify patients at risk of poor response, and the underlying mechanisms, may enable development of improved vaccine strategies for select populations. In only one of the five studies, immune response was studied together with vaccine efficacy in the same patients. Patients with confirmed influenza in the vaccinated group proved to be non-responsive to influenza vaccination [35].

Influenza vaccination had an acceptable safety profile in patients with COPD. There was no consistent evidence that vaccination was associated with reduced lung function or an increased risk of exacerbations in the weeks following vaccination.

Seven studies assessed efficacy/effectiveness of influenza vaccination in preventing adverse clinical outcomes or deaths in patients with COPD using a diverse range of study designs. These studies provided contrasting results linked to different patient characteristics and outcomes studied. Six of the seven studies showed a potential benefit of vaccination over no vaccination. The results of three studies that utilised information from large national health databases showed that influenza vaccination significantly reduced all-cause mortality, deaths associated with a respiratory event and episodes of acute coronary syndrome in patients with COPD, as well as heart failure in persons ≥ 65 years of age. The latter two results suggesting there might be a relation between influenza infection and acute coronary syndrome and heart failure, as is theorized in other studies [49]. Smaller cohort studies showed that influenza vaccination significantly reduced the risk of ARIs and hospitalisation due to COPD exacerbations, as well as the number of outpatient visits. It is worth noting that in one of these studies conducted in Thailand where influenza vaccine had not been previously available, two vaccine doses were administered [35, 40]. It is not certain whether these results are directly applicable to countries where influenza vaccines have been in use for decades, and where a single dose is routinely administered.

Although there was one prospective study that showed no evidence that influenza vaccination reduced deaths in patients with COPD, the results overwhelmingly support a beneficial effect of influenza vaccination on clinical outcomes in patients with COPD. With a mean patient age of 65 year or older in most studies, it is not known whether the benefits of influenza vaccination can be extended to younger patients. Furthermore, efficacy/effectiveness studies were conducted over a period of 15 years. The influenza strains contributing to influenza epidemics vary annually [50], influencing the severity and length of each season. Influenza vaccine components are reviewed annually and the included strains may be updated on WHO recommendations to match the new circulating strains according to the evolution of influenza viruses. Depending on the degree of similarity or difference between the circulating viruses and the strains included in the vaccines, a mismatch can occur, impacting the seasonal influenza vaccine effectiveness [50]. The impact of vaccination is greatest in well matched seasons, and lowest in poorly matched seasons or so called mismatched seasons [50]. However, most of the studies that evaluated efficacy or effectiveness outcomes were conducted in moderately severe influenza seasons with moderate-to-good matches between circulating strains and vaccine strains. One study which segregated the results according to the level of matching between circulating and vaccine strains found that mortality rates were higher in years when the influenza vaccine did not include all strains circulating during that season (RR 1.19, 95% CI 1.13–1.25) [42].

Other potential limitations of this review include that only 13 studies were identified, of which almost one half (6/13) were assessed as being of low quality. Few studies addressed each outcome of interest (immunogenicity, safety or reactogenicity, efficacy, effectiveness), only one study was found that assessed the cost-effectiveness of influenza vaccination, and none assessed the impact on quality of life in this population. During study selection no limits were set on sample size which meant that some studies with relatively low sample sizes, and hence lacking statistical power to be conclusive, were included. In contract to RCTs, in observational studies the patient groups with and without vaccination might differ significantly in characteristics (such as age, comorbidities or COPD severity). Adequate statistical methods should be used to minimize the effects of these differences. However, residual confounding cannot be completely ruled out in observational studies.

Strengths of this review include the focus on studies that specifically assessed COPD and not mixed chronic conditions, the inclusion of immunogenicity data in COPD patients, and the inclusion of observational studies that contribute to understanding the impact of influenza vaccination on long term clinical outcomes including exacerbations, hospitalisations and deaths.

Since the date of our search we have identified two additional articles of interest. A retrospective cohort study of 899 patients with COPD in Spain reported by Garrastazu et al., [14] indicated that influenza vaccination significantly reduced the risk of severe (hospitalised) exacerbations in the year following immunisation (OR 0.54 (0.35–0.84)), with a greater effect in those patients with more severe COPD. Lall et al. [51] published a literature review summarising effectiveness data until June 2014 for influenza immunisation in patients with COPD living in low and middle-income countries and concluded that influenza immunisation was beneficial in these regions. While our review and that of Lall et al., are complementary, they differ in terms of the search dates, such that we were able to include several later studies, and in the population studied; in contrast with our specific focus on patients with COPD, Lall et al., included populations in which up to 90% of individuals did not have COPD.

Despite longstanding recommendations for seasonal influenza vaccination for high risk patients including those with chronic respiratory disease, vaccination rates remains below target levels. ECDC reported seasonal influenza vaccination coverage in patients with chronic medical conditions in 2012–2013 ranging from 28% in Portugal to 80% in the UK and Northern Ireland [9]. In the US, the coverage rate among adults 18–64 years with at least one selected high-risk condition (asthma, diabetes or heart disease) was estimated to be 48% in 2014–2015 [15]. Few data specifically report on influenza vaccine coverage in patients with COPD. A study in Spain showed a seasonal influenza vaccination coverage rate of 60% in 2010 in a population of patients aged ≥40 years with COPD [52]. Targets for seasonal influenza vaccination coverage in at-risk groups are 75% in EU countries [9] and 90% for non-institutionalised high-risk adults 18–64 years of age in the US [53]. Remaining uncertainties about the perceived risk of influenza vaccine-induced exacerbations, the lack of awareness of influenza-associated complications, and the variability of influenza vaccine effectiveness from year-to-year, may contribute to the under-use of influenza vaccine in high risk groups such as those with COPD despite existing supportive recommendations [43].

## Conclusion

Prevention of exacerbations of COPD caused by influenza infections is important for patients with COPD. Seasonal influenza vaccination is recommended by international and national health organisations; nonetheless coverage remains sub-optimal compared to recommended targets. Although we identified a limited number of well-designed, adequately powered and comparable studies aiming to evaluate seasonal influenza vaccine in patients with COPD, the studies supported current recommendations and indicated a positive benefit-risk ratio of vaccination in this population. The available data support annual influenza immunisation of patients with COPD. Knowledge gaps remain in the impact of disease severity and co-morbidity on influenza vaccine effectiveness [54]. Additional large and well-designed observational studies would contribute to a better understanding of the impact of disease severity and patient characteristics on the response to influenza vaccination.

## Additional files

Additional file 1: Search strategy. (XLSX 33 kb)

Additional file 2: Summary tables Safety results. **Table S1** COPD Exacerbations after seasonal influenza vaccination. **Table S2** Spirometric results before, 1 week and 3–4 weeks after seasonal influenza vaccination. **Table S3:** Local and systemic effects after seasonal influenza vaccination (most frequent effects). (XLSX 93 kb)

## Abbreviations

ARI: Acute respiratory illness
CBER: Center for biologics evaluation and research
CHMP: Committee for medicinal products for human use
CI: Confidence interval
COPD: Chronic obstructive pulmonary disease
ECDC: European centre for disease control and prevention
EU: European union
HI: Haemagglutination inhibition
ICD-9: International statistical classification of diseases and related health problems, ninth edition
OR: Odds ratio
PRISMA: Preferred reporting items for systematic reviews and meta-analysis
QIVs: Quadrivalent vaccines
RCTs: Randomised controlled trials
RR: Risk ratio
THIN: The health improvement network
TIVs: Trivalent vaccines
UK: United Kingdom
US: United States
WHO: World health organization
